# Developing and piloting a well-being program for hospital-based physicians

**DOI:** 10.1007/s40037-020-00600-5

**Published:** 2020-07-28

**Authors:** Maarten P. M. Debets, Kiki M. J. M. H. Lombarts, Nathalie I. R. Hugenholtz, Renée A. Scheepers

**Affiliations:** 1 Research Group Professional Performance and Compassionate Care, Department of Medical Psychology, Amsterdam UMC, University of Amsterdam, Amsterdam, The Netherlands; 2 Consumer Safety Institute, Amsterdam, The Netherlands; 3 Research Group Socio-Medical Sciences, Erasmus School of Health Policy and Management, Erasmus University of Rotterdam, Rotterdam, The Netherlands

**Keywords:** Physician well-being, Well-being program, Working conditions, Team-based interventions

## Abstract

**Background:**

Demanding working conditions in medical practice pressurise the well-being of physicians across all career stages, likely harming patients and healthcare systems. Structural solutions to harmful working conditions are necessary as well as interventions to support physicians in contemporary practice. We report on developing and piloting a team-based program for physicians to improve their working conditions and well-being.

**Approach:**

Program development steps involved: a preparatory phase, needs assessment, and program design. The program consisted of (1) a feedback tool addressing working conditions and well-being, and an intervention including (2a) a facilitated team dialogue and (2b) a team training on communication and collaborative job crafting. In the program’s pilot, 377 physicians from 48 teams in 14 Dutch hospitals used the feedback tool. Four teams participated in the team dialogue. Two teams performed the team training.

**Evaluation:**

Physicians indicated that the program was a useful format to gain insight into their working conditions and well-being, and possibly to improve their well-being collaboratively.

**Reflection:**

We provide seven critical reflections on developing and piloting our program, accompanied by recommendations for developing well-being interventions. Our development approach, program components, and recommendations may support physicians and other healthcare professionals in demanding work environments.

## Background and need for innovation

Physicians’ well-being is essential for delivering high-quality patient care [1–3]. Unfortunately, the well-being of medical students and physicians across all career stages is at considerable risk, evidenced by many studies showing high levels of burnout [4, 5]. Recently, a meta-analysis associated physician burnout with increased odds of unsafe patient care, unprofessional behaviours, and lower patient satisfaction [3]. Burnout is a result of chronic workplace stress that has not been successfully managed [6]. Still, most interventions addressing burnout in healthcare have focused on enhancing individuals’ stress management skills [7, 8].

Equipping individuals with stress management skills can be helpful but does not provide a structural solution to workplace stressors [3, 9]. The overrepresentation of individualistic approaches is, however, not surprising. Adjusting working conditions in hospitals is often more complex, costly, and out of the control of individual physicians or even teams [9]. Nonetheless, if physicians within a team discuss their experiences about the workplace and their well-being, together they might be able to recognise and address workplace stressors and resources [9–11]. Moreover, physicians might assist each other in finding solutions to occupational well-being issues. Literature indicates that teams proactively reducing stressors and improving resources in the workplace are more engaged and perform better [11].

Therefore, we developed a team-based well-being program for hospital-based physicians. In this study, we reflect on developing and piloting the program. This contribution aims to inform healthcare professionals and intervention developers, and support them in improving physicians’ well-being.

## Goal of innovation

The goal of our program was to assist physicians in improving their working conditions and well-being.

## Steps taken for development and implementation

The Dutch Ministry of Social Affairs and Employment funded the program development. Following the approved grant proposal, the program included (1) a feedback tool addressing working conditions and well-being, and (2) a team-based intervention aimed at improving working conditions. The project team consisted of researchers (MD, KL, RS), trainers (NH), and software developers, all familiar with the medical profession.

The project team developed the program in three consecutive steps: a preparatory phase, needs assessment, and program design (October 2016 until March 2017). Next, we piloted the program (April 2017 until September 2017).

The institutional ethical review board of the Academic Medical Center of the University of Amsterdam (AMC) confirmed that the Medical Research Involving Human Subjects Act (WMO) did not apply to this study and thus waived ethical approval (reference number: W18_234 # 18.279).

### Step 1—Preparatory phase

Two researchers searched and mapped reliable and valid measures of physicians’ working conditions and well-being for potential inclusion in the feedback tool. To inform the intervention, they also mapped evidence-based interventions to improve physicians’ well-being. We used the resulting overview to construct the needs assessment’s survey.

### Step 2—Needs assessment

Our needs assessment included one focus group in an academic hospital (*n* = 12) and one in a non-academic teaching hospital (*n* = 12), followed by an online survey (*n* = 218).

The focus groups lasted 75 min and aimed to obtain in-depth insight into physicians’ needs concerning their working conditions and well-being. Beside residents and medical specialists, we invited human resources staff and senior hospital management to illustrate how hospital policies and practices could address physicians’ needs. Four key questions structured the discussion: ‘What characterises well-being in practice?’, ‘What needs do physicians have to improve their well-being?’, ‘What influences physicians’ well-being in practice?’, and ‘What possibilities do you see for promoting physicians’ well-being?’. A moderator facilitated the focus groups; two observers made notes about verbal and non-verbal communication. Participants indicated that a feedback tool to assess working conditions and well-being should be easily accessible, time-friendly, and encourage discussion. Furthermore, an intervention should provide a positive and psychologically safe environment. Also, it should address team members’ shared workplace issues (e.g. lack of social support) while respecting individuals’ needs (e.g. no collegial contact outside working hours).

The survey aimed to quantify physicians’ needs regarding the feedback tool and intervention. Using the previously mentioned overview (step 1), we listed working conditions and well-being aspects for which validated measures were available, as well as evidence-based interventions. The survey asked physicians to rate working conditions (e.g. workload) and well-being aspects (e.g. work engagement) of interest. Additionally, physicians indicated preferred methods of discussing the feedback tool’s results and evidence-based interventions.

Project team members invited physicians from Dutch hospitals for the survey using their professional networks, company newsletters and websites. In total, 218 physicians participated, of which 50.3% were male. The mean age was 43.3 (*SD* = 9.97) years. The most represented specialties were surgery (17.8%), neurology (14.1%), and internal medicine (12.0%). Of the working conditions of interest, administrative burden, appreciation by patients, learning and professional development opportunities, inspirational leadership, and workload were most frequently rated. The top rated well-being aspect was work-life balance. Furthermore, physicians preferred to discuss the feedback tool’s results in a facilitated team dialogue. The most preferred interventions were team communication training and collaborative job crafting training.

### Step 3—Program design

**Fig. 1:**
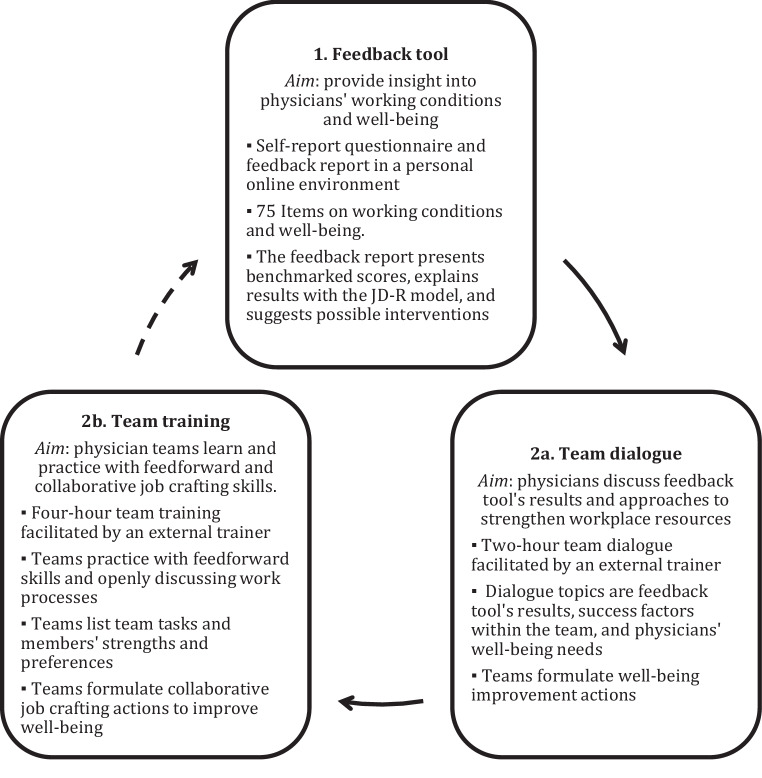
The well-being program for physicians and its components. The *dotted arrow* depicts the suggested continuous approach to improve working conditions and well-being, although in this project usage of the feedback tool was not repeated

Based on the previous steps, we designed the content of the (1) feedback tool and (2) intervention, shown in Fig. 1. The job demands-resources (JD-R) model [12] and positive psychology [13] guided the program design. According to the JD‑R model, individuals classify perceived working conditions as job demands (i.e. workplace stressors, requiring energy) or job resources (i.e. workplace resources, providing energy). Optimising the balance between both can improve well-being. Also, focusing on enhancing team strengths and workplace resources—i.e. positive psychology—is worthwhile to improve well-being [14]. Workplace resources are functional in achieving work goals, stimulating personal growth, and alleviating the negative impact of stressors [12, 14].

The feedback tool (1) consisted of a self-report questionnaire and feedback report on perceived working conditions and well-being. The questionnaire counted 75 items (completion time of 10–15 min) based on validated measures of working conditions (i.e. administrative burden, collegial support, inspirational leadership, intrinsic motivation for patient care, learning and professional development opportunities, participation in decision making, workload) and well-being (i.e. emotional exhaustion, work engagement, work fatigue, work-home interference). Software developers implemented the feedback tool in an existing online environment, wherein physicians could conduct the questionnaire and download the feedback report. The feedback report included results benchmarked against ratings from peers and explained the JD‑R model assisting physicians to analyse working conditions in relation to their well-being. When peer scores were unavailable, the report showed benchmarks of the general working population.

The intervention consisted of two consecutive parts: (2a) a facilitated team dialogue and (2b) a team training on team communication and collaborative job crafting (Fig. 1). The (2a) team dialogue was a two-hour session led by an external trainer, in which teams discussed their working conditions and well-being to formulate improvement actions. The feedback tool’s results served as input for the dialogue, although physicians decided what they wanted to share. We organised a focus group with trainers and senior physicians (*n* = 8) to design a team dialogue guide.

This guide included an exemplar schedule and defined preferred conditions for a productive meeting (i.e. no beepers, external facilitator). Also, the guide suggested an appreciative inquiry approach, which invites participants to discuss stories about what is working well. The resulting identified strengths are the starting point for positive change actions [15].

The (2b) team training was a four-hour training in which physicians practised with providing team members feedforward. Furthermore, physicians exercised collaborative job crafting to address the formulated improvement actions regarding working conditions and well-being from the facilitated team dialogue. Typically, feedforward focuses on future expectations and tasks to create lasting improvement [16]. Collaborative job crafting refers to physicians determining together how to alter workplace stressors and resources to meet their well-being goals [11]. To design the team training, trainers used prior communication and job crafting workshops, and collaborative job crafting literature [17]. The training combined both topics because of the relevance of communication for team learning of job crafting [17].

### Pilot testing

Project team members invited physician teams to participate in the program’s pilot using their professional networks, company websites and newsletters. We piloted the feedback tool in 14 Dutch hospitals, and 377 physicians from 48 teams completed the questionnaire (71% response rate) and received a feedback report: 47.7% were male, 78.8% medical specialists, and 21.2% residents. After completing the feedback tool, teams were more inclined to participate in the facilitated team dialogues. We selected physician teams based on variation in size and specialty (medical or surgical) and conducted four team dialogues in different hospitals. From those teams, two were willing and available to address their formulated improvement actions in the team training, completing all program components.

To evaluate the program, we inspected respondents’ answers on open text evaluations of the feedback tool and consulted its helpdesk to obtain insight into participants’ experiences. Furthermore, we examined observers’ notes from the intervention, inspected printed evaluation forms from the team training, and conducted 14 telephone interviews with participants.

## Evaluation of innovation

Physicians perceived the program as a useful approach (Fig. 1) to address working conditions, and presumably to improve well-being. In the following sections, we report on the evaluation of each program component.

### Feedback tool

According to the participants, the feedback report provided insight into their working conditions and well-being by explaining their benchmarked questionnaire results. During the pilot, we used anonymised results of the feedback tool to calculate more specific peer benchmarks for all measures. Physicians evaluated the feedback tool’s online environment as accessible and user-friendly. The questionnaire items represented physicians’ work well, although some were open to multiple interpretations. For instance, concerning inspirational leadership items, some self-employed physicians mentioned not having a direct supervisor. To improve the questionnaire, participants suggested to include more items about their personal life, a ‘not applicable’ option, and ‘free text boxes’ to clarify answers.

### Intervention

Physicians appreciated the facilitated team dialogues’ structured and theory-based approach. During the sessions, physicians shared knowledge and reflected on their personal qualities, team strengths, and coping with workplace stressors, leading to practical well-being improvement actions. For example, team members discussed a rotation system to attend management meetings, joint administration days, and taking small breaks in the ambulance hall. Physicians valued the opportunity for team-based reflection on working conditions and well-being since this was uncommon in clinical practice.

During the team training, most physicians experienced practising feedforward and collaborative job crafting positively. They mentioned obtaining insight into their own and colleagues’ strengths and task preferences, and exchanged tasks accordingly. Also, physicians appreciated job crafting examples of other teams. However, some were sceptical about collaborative job crafting, as they believed there is not much to ‘craft’ in the medical environment due to regulations and focus on production quotas. Finally, physicians regarded the four-hour duration as a barrier for scheduling and participating in the training, especially given the team-based approach.

## Critical reflections

In this section, we provide seven critical reflections on the development, implementation, and evaluation of our well-being program, accompanied by recommendations for developing well-being interventions (Tab. 1).

**Table 1:** Recommendations for designing well-being interventions for physicians based on seven critical reflections

Development	1. Involve physicians and stakeholders with diverse backgrounds
	2. Consider the implications of proposed recruitment and selection strategies concerning interventions’ goals
Implementation	3. Align well-being interventions with existing (online) infrastructure
	4. Facilitate physicians while engaging in well-being interventions
	5. Create a psychologically safe environment for physicians
Evaluation	6. Consider rigorous evaluation of implementation strategies and interventions’ effectiveness timely
	7. Design interventions with a continuous character, using instruments to stimulate discussion and measure change

### Development

First, involving physicians was essential for gaining insight into physicians’ needs and aligning the program with the medical work environment. Also, we found that involving researchers, trainers and software developers was valuable as they each offered unique expertise during project team meetings, jointly developing a well-integrated program serving both user-friendliness and scientific robustness. We recommend involving physicians and stakeholders with diverse backgrounds when developing well-being interventions.

Second, we recommend using recruitment and selection strategies corresponding to interventions’ goals. Our goal was to assist physician teams willing to improve their working conditions and well-being. Therefore, we aimed at recruiting these teams by employing various strategies, such as using company newsletters. Perhaps, physicians interested in improving working conditions and well-being were more inclined to participate, possibly leading to an overrepresentation of their voice [18]. Consequently, our program and the pilot results might not apply to all physicians. Different recruitment strategies might be needed to reach those less affiliated with our program’s goal. However, based on the diversity of participating teams, we do not have the impression that specific subgroups were overrepresented.

### Implementation

Third, physicians already familiar with the online environment accommodating the feedback tool needed less (technical) support than those using other web-based platforms. Employing one online system for multiple performance assessments enabled more natural coordination for planning assessments, limiting the chance of measurement fatigue. Accordingly, we recommend aligning well-being interventions with existing (online) infrastructure.

Fourth, we recommend facilitating physicians engaging in well-being interventions, as it might prevent experiencing participation as yet another job demand. Multiple methods can foresee in offering support, such as having a helpdesk, scheduled participation time and no participation fees.

Fifth, from the project’s start, we realised that psychological safety was crucial, yet not self-evident, for a team-based well-being intervention. We recommend creating a psychologically safe environment for participants, for example by adopting a leadership style in which every team member gets heard and acknowledged [19]. Our program facilitated physicians’ psychological safety by anonymous assessment, offering aftercare, using experienced external trainers, focusing on working conditions in team dialogues, and using appreciative inquiry as a strengths-based approach.

### Evaluation

Sixth, the one-year time frame, defined by the grant proposal, left no time to evaluate the program’s effectiveness more deeply nor to implement it in other hospitals. We recommend considering timely and rigorous evaluation of implementation strategies and interventions’ effectiveness. Although we did not test our program’s effectiveness over time, our evaluation methods provided insight into participants’ experiences and the functioning of program components.

Seventh and lastly, this project showed the value of a continuous approach (Fig. 1) to improve working conditions and well-being. The feedback tool—potentially serving as an intervention itself—stimulated participation and discussion in team dialogues, leading to actions to address in the training. Moreover, the feedback tool can repeatedly measure changes in workplace conditions and well-being, providing novel input for enhancement while also monitoring the program’s effectiveness. Therefore, we recommend designing well-being interventions with a continuous character using instruments sensitive to change.

## Conclusion

In response to work-related risks to physicians’ well-being, we successfully developed and piloted a program addressing physicians’ working conditions and well-being. We reported and reflected on our approach and provided recommendations for developing similar interventions—hopefully contributing to the improvement of physicians’ workplaces and health.
